# Posterior versus lateral surgical approach: functionality and quality of life after total hip arthroplasty in a matched cohort study

**DOI:** 10.1186/s12891-021-04679-7

**Published:** 2021-11-08

**Authors:** Davide Castioni, Olimpio Galasso, Bruno Iannò, Michele Mercurio, Giorgio Gasparini

**Affiliations:** 1 Department of Orthopaedic and Trauma Surgery, “Magna Græcia” University, “Mater Domini” University Hospital, V.le Europa, (loc. Germaneto), 88100 Catanzaro, Italy; 2 Department of Surgery, Division of Orthopedics and Trauma Surgery, “G. Jazzolino” Hospital, Piazza Fleming, 89900 Vibo Valentia, Italy

**Keywords:** Total hip arthroplasty, Surgical approach; health-related quality-of-life, Complications, Trendelenburg; residual pain, Serum creatine phosphokinase, Activities of daily living, WOMAC, SF-36

## Abstract

**Background:**

One of the most controversial aspects for maximizing outcomes after total hip arthroplasty (THA) remains the surgical approach to the hip joint. The posterior (PA) and lateral approaches (LA) are the two most commonly performed approaches used worldwide, but sparse data are available for their comparison in terms of health-related quality-of-life (HRQoL). The aim of this study was to assess the role of the PA and LA in the HRQoL and hip functionality of patients who underwent primary and elective THA for osteoarthritis, after a minimum 2-year follow-up.

**Methods:**

One hundred twenty-eight patients (140 THAs: 68 with PA and 72 with LA) were evaluated in a matched cohort study. Data gathered included the body mass index, the American Society of Anesthesiologists score, surgery time, serum creatine phosphokinase (CpK) levels, estimated intraoperative blood loss and intra- or postoperative complications. Preoperatively and at the last follow-up, the activities of daily living, and the instrumental activities of daily living (IADL) scales, the Western Ontario and Mac Master University (WOMAC) Questionnaire, the Harris Hip Score (HHS) and the Visual Analogue Scale (VAS) were used to assess HRQoL and functionality. The Short Form-36 Health Survey (SF-36) Questionnaire was administered at the last follow-up.

**Results:**

Postoperatively, CpK was higher in the LA group compared to the PA (695 ± 648 vs. 447 ± 326 UI/L, *p* < 0.001). At a mean follow-up of 47 ± 22 months for the LA group and 42 ± 29 months for the PA group, IADL, VAS, HHS and WOMAC scores significantly improved for both groups (all *p* < 0.001), but PA reported better VAS, residual pain and WOMAC scores (*p* = 0.002, *p* = 0.004 and *p* = 0.018, respectively). The PA group demonstrated a significant higher mental SF-36 subscale values than the LA group (49 ± 13 vs. 42 ± 19, *p* = 0.001). The LA group showed a higher number of Trendelenburg signs (*p* = 0.029). On the contrary, the PA group showed a higher number of leg lengthening (*p* = 0.020); however, most of these cases was less than the clinically significant value of 10 mm (*p* = 0.738).

**Conclusions:**

Patients who underwent THA performed with the PA reported greater improvement in HRQoL with lower residual pain, postoperative muscle damage and Trendelenburg signs than those who underwent the LA.

## Background

Total hip arthroplasty (THA) is a successful and safe procedure for managing patients with disabling articular pain and functional limitations caused by a number of diseases, such as osteoarthritis (OA), inflammatory arthritis, avascular necrosis, and fractures.

Despite these excellent results, after THA, from 7 to 15 % of patients report dissatisfaction [1–3], posing a challenge to physicians. The muscle damage associated with surgery, restoration of function, relief from pain and quality of life are critical for maximizing outcomes [1, 4].

In this light, one of the most controversial aspects remains the surgical approach to the hip joint. Different approaches have been proposed and refined, including anterior, anterolateral, lateral, and posterior approaches. The posterior approach (PA) and the lateral approach (LA) are the two most commonly performed approaches used worldwide [5]. The PA is associated with fewer problems regarding gait since the abductor is not dissected; however, there is a risk of damage to the sciatic nerve during dissection or compression with retractors, as it lies over the overturned external rotator muscles of the hip. In the PA, cup positioning is often more difficult; thus, increased rates of dislocation have been reported in comparison with the LA [6]. Moreover, if the external rotators and the capsule are not repaired, instability rates can be increased. In contrast, the LA is associated with injury to the superior gluteal nerve and heterotopic ossifications (HOs). Systematic reviews examining the comparison of the PA and LA for THA have been published with specific regard to complications and functional outcomes [6, 7]. However, sparse data are available for the comparison of these surgical approaches in terms of health-related quality of life (HRQoL) [8–11]; only one study [11] assessed HRQoL using the Short Form-36 Health Survey (SF-36), but the assessment was performed at a short-term follow-up of 12 weeks.

The aim of this study was to assess the role of the PA and LA in the HRQoL and hip functionality of patients who underwent THA after a minimum 2-year follow-up.

## Methods

A retrospective matched-cohort study was performed with collection of prospective data on 150 patients who underwent primary THA for OA at our institution between January 2008 and January 2018. The study protocol was approved by the local ethics committee, and the research was conducted in compliance with the Declaration of Helsinki. Informed consent was obtained from all individual participants included in the study. The inclusion criteria were (1) primary and elective THA for OA, (2) surgery performed using the PA or LA and (3) participation in a minimum 2-year follow-up. The exclusion criteria were (1) conversion THA with removal of hardware, (2) revision THA, (3) THA performed for any diagnosis other than primary OA, (4) concomitant neurological and orthopedic diseases of the lower limbs and (5) failure to understand or complete the questionnaires. Twelve patients died of causes unrelated to the procedure, and 10 did not participate in a follow-up; therefore, 128 of 150 patients (140 THAs: 68 with the PA and 72 with LA) were enrolled and evaluated.

Data gathered included the age of the patient, body mass index (BMI), American Society of Anesthesiologists (ASA) score, surgery time, estimated intraoperative blood loss (EBL) using the following formula: [EBL= (preoperative hemoglobin (Hb) – postoperative Hb)/preoperative Hb x 100] [12], and intra- or postoperative complications.

To assess muscle damage, serum creatine phosphokinase (CpK) levels were measured preoperatively and 48 h after surgery when the maximum increase in values was expected [13]. It has been reported that after appropriate correction for BMI, an increase in CpK level following trauma, exercise, or surgery directly correlates with muscle damage, resulting in an objective method with which to evaluate the impact of surgical approaches in arthroplasty [14].

### Surgical technique

All surgical procedures were performed by three surgeons (GG, OG and BI) with high levels of experience in hip arthroplasty. All patients were placed in the lateral decubitus position, which was carefully checked to ensure the pelvis was perpendicular to the ground. Deep vein thrombosis (DVT) prophylaxis was carried out by administration of low-molecular-weight heparin [15], antibiotic prophylaxis was administered intravenously as recommended [16], and in the absence of contraindications, either spinal or epidural anesthesia was performed for all procedures.

The PA was described by Gibson [17] and popularized by Moore in the 1950 s [18]; this approach employs superficial plane splitting through the gluteus maximus and requires a tenotomy of the short external rotators of the hip and a posterior capsulotomy. The femoral head is then dislocated by internally rotating the hip, and the neck is osteotomized.

The LA described by Hardinge in 1982 has undergone numerous modifications [19, 20]. This approach makes use of the superficial interval between the tensor fasciae latae and gluteus maximus. In addition, the gluteus medius and vastus lateralis are split in continuity, leaving a cuff of the gluteus medius tendon for repair following the procedure. The gluteus minimus and joint capsule are incised in line with the neck of the femur. The femoral head is then dislocated by externally rotating and flexing the hip and knee.

In this study, regardless of the approach used, all THAs were performed using a cementless, proximally hydroxyapatite-coated, tapered stem with a cementless, hemispherical acetabular shell, a ceramic femoral head, and a polyethylene acetabular liner (*Corail*® femoral stems and *Pinnacle*® acetabular component; *DePuy International Ltd*, Leeds, England). A closed-suction drain was applied and removed within 24 h.

Postoperatively, no specific prophylaxis against HOs was performed. A multimodal analgesia strategy combining an intravenous formulation of acetaminophen (1 g every 12 h for 5 days), an injectable nonsteroidal anti-inflammatory drug (i.e., diclofenac 75 mg every 12 h for 4 days), and an oral opioid (i.e., tapentadol 50 mg every 12 h for 3 days) was used in the absence of specific contraindications to improve postoperative pain and to reduce the consumption of each agent. Postoperatively, patients underwent early weight-bearing the same day of surgery and mobilization and ambulation with walking aids and muscle strengthening exercises after the removal of the drain.

### Functional independence and health-related quality of life assessment

Preoperatively and at the last follow-up, each patient was evaluated with the activities of daily living (ADL) and the instrumental activities of daily living (IADL) scales and the Italian version of the Western Ontario and McMaster Universities (WOMAC) questionnaire [21–23].

The term ADL was first coined by Katz in 1950 [21], and it is used to describe the collective fundamental skills that are required for independent self-care. Katz’s ADL scale is used to measure six tasks in hierarchical order of decreasing difficulty as follows: bathing, dressing, toileting, transferring to and from a chair, maintaining continence and feeding. Responses to each of the six tasks in the scale are coded as 0 (dependent) or 1 (independent), and the responses are summed. A score of 6 points indicates independent functioning, and a score of 0 points indicates full dependence.

The IADL is used to assess different activities than those assessed with the ADL, with the former measuring the activities that allow an individual to live independently in a community, thereby improving quality of life. Lawton’s IADL Scale [22] is composed of 8 items and assesses a person’s ability to perform tasks such as using a telephone or handling finances. Responses to each of the eight items in the scale are coded as 0 (unable or partially able) or 1 (able), and the responses are summed. The summary score ranges from 0 (low functioning, dependent) to 8 (high functioning, independent).

The WOMAC questionnaire is a self-administered disease-specific validated outcome measure, and it was used to assess pain, stiffness, and physical function disability in patients suffering from knee and hip osteoarthritis [23]. The WOMAC questionnaire provides either single domain scores or a total score (0–100); lower scores are associated with less pain and stiffness and better function.

At the last follow-up, the Italian version of the SF-36 questionnaire [24] was also assessed. The SF-36 is a generic measure of health status that contains 36 questions measuring the physical, social, and mental components of respondents. This questionnaire yields an eight-scale profile of scores (i.e., physical functioning, PF; role physical, RP; bodily pain, BP; general health, GH; vitality, VT; social functioning, SF; role emotional, RE; and mental health, MH) as well as physical component summary (PCS) and mental component summary (MCS) measures. The SF-36 results were compared to normative data [24, 25].

### Functional assessment

Preoperatively and at the last follow-up, each patient was evaluated with the Harris Hip Score (HHS) and the Visual Analog Scale (VAS).

The HHS is a disease-specific test to evaluate hip disability [26]; scores range between 0 and 100 and include evaluations of pain, function, deformity, and motion domains. A total score of < 70 was considered a poor result, 70–79 fair, 80–89 good, and 90–100 excellent [20]. To assess the degree of HHS improvement, a recovery rate (RR) was computed utilizing the following formula: [RR = (postoperative value – preoperative value)/postoperative value x 100] [27].

The VAS was used as a subjective measure of pain perception. A VAS score > 3 at the last follow-up indicated residual pain [28]; THAs with known causes of pain (e.g., infection, stiffness or loosening, instability, a fracture or neurovascular injury, or comorbidities) were excluded for the purpose of residual pain analysis only.

Leg length discrepancy (LLD) was assessed using X-rays film showing standing full-length AP of both lower extremities. As previously reported, we considered a clinically significant LLD when shortening exceeds 10 mm and lengthening exceeds 6 mm[29]. The presence of the Trendelenburg sign was also clinically evaluated. Preoperative and postoperative patient assessments were performed by trained physicians (DC and MM) who were not involved in the primary care of the patient.

### Statistical analysis

All data were measured, collected, and reported to one-decimal accuracy. The mean, standard deviation, and range were noted for the continuous variables, counts for the categorical variables were recorded. The distribution of the numeric samples was assessed by Kolmogorov-Smirnov normality test. Based on this preliminary analysis, parametric tests were adopted. To evaluate the significance of differences between preoperative and postoperative values, a two-tailed paired-sample Student’s t-test was performed.

IBM SPSS Statistics software (version 26, IBM Corp., Armonk, NY, USA) and G*Power (version 3.1.9.2, Institut für Experimentelle Psychologie, Heinrich Heine Universität, Düsseldorf, Germany) were used for database construction and statistical analysis. A p-value of less than 0.05 was considered significant.

## Results

Seventy-two (51.4 %) hips underwent THA through the LA, and the PA was used in 68 operations (48.6 %). Table 1 shows and compares the demographics and characteristics of the included cases at baseline. No significant differences were found for most of the variables between the two groups; the PA group presented a significantly higher preoperative ASA score (*p* = 0.030) than the LA group. The mean operation time was 148.5 ± 30.4 min for the LA group and 132.6 ± 29.9 min for the PA group (*p* = 0.002). In our study, we also considered the preoperative and postoperative values ​​of some laboratory parameters. The measurements of EBL were not different between the LA and PA groups (30.1 %±8.1 and 33.2 %±10.9 %, respectively); however, a trend indicated that the LA group had a higher EBL score (*p* = 0.059). For the LA group, serum CpK levels increased from 117.7 ± 68.9 to 694.6 ± 647.6 UI/L; for the PA group, they increased from 116.5 ± 69.3 to 447.4 ± 325.5 UI/L. This increase was expected, with peaks on the second postoperative day for both the LA and PA groups (*p* < 0.001). Although no preoperative differences between the two groups were noticed, postoperative CpK values were significantly higher in the LA group than in the PA group (*p* = 0.049).

**Table 1:** Demographics of lateral and posterior approach groups

		LA *mean* ± *SD or % (No.)*	PA *mean* ± *SD or % (No.)*	*p-value*
operation		51.4 % (72)	48.6 % (68)	*ns*
age *(years)*	65.3 ± 8.8		68.1 ± 7.8	*ns*
gender *M:F*	54 %:46 % (39:33)		49 %:51 % (33:35)	*ns*
BMI *(kg/m2)*				
preoperative	29.4 ± 6.0		29.3 ± 6.2	*ns*
* follow-up*	29.2 ± 5.4		29.3 ± 5.8	*ns*
* p-value*	*ns*		*ns*	
side				
right	61.1 % (44)		63.2 % (43)	*ns*
left	38.9 % (28)		36.8 % (25)	*ns*
ASA score [*1-5*]	2.0 ± 0.8		2.3 ± 0.8	***0.030***

*LA* means lateral approach, *PA* posterior approach, *SD* standard deviation, *M* male, *F* female, *BMI* body mass index, *ASA* American Society of Anesthesiologists grade, *ns* not significant

Table 2 shows the differences between ADL, IADL, VAS, HHS and WOMAC scores before surgery and at follow-up. At a mean follow-up of 46.8 ± 22.2 months for the LA group and 42.1 ± 28.7 months for the PA group (*p* = 0.279), the preoperative ADL score had significantly improved at the follow-up only in the PA group (*p* < 0.001). IADL, VAS, HHS and WOMAC scores significantly improved at the follow-up for both the LA and PA groups (all *p* < 0.001). Specifically, the improvement in HHS values was 57.5 ± 17.9 with an RR of 379 % for the PA group and 49.0 ± 19.3 with an RR of 203 % for the LA group.

**Table 2:** Differences in scores over time and between the two groups

	LA (patients = 72) *mean* ± *SD*	PA (patients = 68) *mean* ± *SD*	*p-value*
preop ADL	5.2 ± 1.2	5.0 ± 1.3	*ns*
ADL at follow-up	5.2 ± 1.3	5.5 ± 1.1	*ns*
*p-value*	*ns*	***< 0.001***	
preop IADL	6.0 ± 1.9	5.6 ± 2.1	*ns*
IADL at follow-up	6.8 ± 1.9	6.8 ± 2.0	*ns*
*p-value*	***< 0.001***	***< 0.001***	
preop WOMAC	70.7 ± 13.6	76.1 ± 14.9	*ns*
WOMAC at follow-up	20.9 ± 20.7	14.5 ± 17.5	***0.018***
*p-value*	***< 0.001***	***< 0.001***	
preop VAS	8.7 ± 1.5	9.2 ± 1.2	*ns*
VAS at follow-up	2.5 ± 2.5	1.3 ± 1.9	***0.002***
*p-value*	***< 0.001***	***< 0.001***	
preop HHS	33.9 ± 15.3	28.5 ± 14.8	***0.036***
HHS at follow-up	82.9 ± 20.3	86.0 ± 16.6	*ns*
*p-value*	***< 0.001***	***< 0.001***	

*LA* means lateral approach, *PA* posterior approach, *SD* standard deviation, *ADL* Activities of Daily Living, *IADL* Instrumental Activities of Daily Living, *WOMAC* Western Ontario and McMaster University, *VAS* Visual Analogue Scale, *HHS* Harris Hip Score, *ns* not significant

At follow-up, we found lower VAS and WOMAC scores in the PA group than in the LA group (*p* = 0.002 and *p* = 0.018, respectively). A VAS score > 3 was noted in 23 cases (32.4 %) in the LA group and in 8 (11.8 %) in the PA group; thus, residual pain was significantly higher in the LA group (*p* = 0.004).

As shown in Table 3, in both the LA and PA groups, the mean SF-36 scores for all eight dimensions were slightly lower than the mean of the age-matched healthy population [24, 25]; the mean SF-36 summary component scores for the LA group ​​were also lower than the normative values: 39.4 ± 13 vs. 42.7 ± 9 for the PCS-36 and 41.9 ± 13 vs. 45.8 ± 9 for the MCS-36. In the PA group, the mean SF-36 PCS ​​was lower than the normative values (42.0 ± 13 vs. 42.7 ± 19), and the mean SF-36 MCS value was higher than the normative values (48.8 ± 13.0 vs. 45.8 ± 9). The PA group demonstrated a significantly higher SF-36 MCS score than the LA group (48.8 ± 13 vs. 41.9 ± 19, *p* = 0.001).

**Table 3:** SF-36 scores among lateral and posterior approach groups, and normative data [25]

SF-36 scale	LA (patients = 72) *mean* ± *SD*	PA (patients = 68) *mean* ± *SD*	population norms^a^* mean ± SD*
PF	49.3 ± 26	61.8 ± 24	*71.7* ± *24*
RP	61.6 ± 40	63.8 ± 40	*65.9* ± *38*
BP	50.8 ± 26	64.5 ± 24	*67.6* ± *26*
GH	54.7 ± 18	60.8 ± 16	*55.4* ± *19*
VT	43.6 ± 19	60.6 ± 18	*59.3* ± *19*
SF	78.7 ± 25	82.4 ± 24	*75.8* ± *23*
RE	61.6 ± 36	72.0 ± 34	*73.5* ± *34*
MH	51.9 ± 21	66.5 ± 18	*64.7* ± *19*
PCS-36	39.4 ± 13	42.0 ± 13	*42.7* ± *9*
MCS-36	41.9 ± 19	48.8 ± 13	*45.8* ± *9*

*LA* lateral approach, *PA* posterior approach, *SD* standard deviation, *SF-36* Short Form-36 Health Survey, *PF* physical functioning, *RP* role physical, *BP* bodily pain, *GH* general health, *VT* vitality, *SF* social functioning, *RE* role emotional, *MH* mental health, *PCS-36* Physical Component Summary, *MCS-36* Mental Component Summary

^a^65–74 years old individuals

Complications are reported in Table 4: a higher number of Trendelenburg signs was noted in the LA group (*p* = 0.029). In contrast, a higher number patients with leg lengthening was noted in the PA group (*p* = 0.020); however, most of the lengthening was less than the clinically significant value (i.e., 12 of 17 patients in the LA group and 22 of 29 patients in the PA group; *p* = 0.738) [29].

**Table 4:** Comparison of complications between the lateral and posterior approach

	LA *(patients = 72) No. (%)*	PA *(patients = 68) No. (%)*	*p-value*
dislocation	1 (1.4 %)	0 (0 %)	*ns*
trochanteric pain	0 (0 %)	2 (2.9 %)	*ns*
Trendelemburg sign	29 (32.2 %)	15 (22.0 %)	***0.029***
aseptic loosening	3 (4.2 %)	1 (1.5 %)	*ns*
periprosthetic fracture	0 (0 %)	0 (0 %)	*ns*
intraoperative fracture	0 (0 %)	0 (0 %)	*ns*
nerve palsy	1 (1.4/%)	1 (1.5 %)	*ns*
infection	0 (0 %)	0 (0 %)	*ns*
postop hematoma	4 (5.6 %)	2 (2.9 %)	*ns*
leg length discrepancy	17 (18.9 %)	29 (42.7 %)	***0.020***
deep vein thrombosis	0 (0 %)	2 (2.9 %)	*ns*

*LA* means lateral approach, *PA* posterior approach; *ns*, not significant

## Discussion

In the current study, after a minimum 2-year follow-up, patients who underwent THA using the PA reported greater improvement in HRQoL as assessed by SF-36 MCS and WOMAC scores and lower residual pain than those who underwent THA with the LA. Higher CpK levels and more patients showing Trendelenburg signs were observed in the LA group after surgery.

The PA and LA are the two most common surgical approaches performed worldwide for THA [5], but a limited number of comparative studies on the PA versus LA [8–11] have evaluated the impact on HRQoL after the index procedure. The development of HRQoL instruments has made it possible to obtain an objective assessment of the impact of surgical procedures taking into consideration the physical, psychological, and social aspects of the patient’s everyday activities. We administered two widely used and validated HRQoL questionnaires, namely, the SF-36 and WOMAC, which have been recommended for studying THA patients [30]; notably, both questionnaires are recommended when different techniques are compared [31]. Postoperatively, we detected greater SF-36 MCS and lower WOMAC scores in the PA group than in the LA group. Witzleb et al. [11] compared the short-term outcomes of THA using both surgical approaches in a randomized controlled trial of 60 patients and found that the SF-36 MCS and PCS and WOMAC scores showed no differences 12 weeks after surgery. Among other general quality of life outcome questionnaires, the EuroQol-5D can be used for evaluating HRQoL in a THA population [32]. Jameson et al. [8] showed that the LA group was associated with significantly lower improvement in the EuroQol-5D index than the PA group.

A meta-analysis revealed that the most effective approach for improving VAS scores was the LA [33]. We noted that the PA group had lower residual pain than the LA group at follow-up. However, the reported significant difference in VAS score between the two approaches (it was higher in the PA group, at 1.2) was lower than the minimal clinically important difference for the VAS score after THA (i.e., 1.9) [34]. Further analysis of the residual pain confirmed better results in the PA group, with only 8 cases (11.8 %) in comparison with 23 cases (32.4 %) in the LA group.

In our sample, the postoperative HHS was comparable between the two approaches even though the LA group showed a higher preoperative HHS. The HHS significantly improved after surgery, with good to excellent clinical results observed in 73.5 and 72.2 % of patients in the PA and LA groups, respectively, and no significant differences were noted between the two groups. Furthermore, the improvement in HHSs was higher for both surgical approaches than the minimal clinically important difference reported by Singh et al. after primary THA [35]. Ji et al. [36] showed similar findings with comparable mean postoperative HHSs between the two approaches.

Gore et al. [37] found reduced abductor muscle strength in the LA group, and Roselund et al. [38] showed that patients in the PA group self-reported fewer Trendelenburg signs at the 12-month follow-up. These findings are in agreement with our results. Bahl et al. [39] suggested that there is a common risk of superior gluteal nerve damage regardless of the surgical approach used, including retraction, direct dissection, compression due to hematoma or scar tissue, and thermal injury. Equal muscle strength between the two approaches was reported by Downing et al. [40] and Kiyama et al. [41]. A Cochrane review based on four nonrandomized cohort studies found no differences in limping between patients who underwent surgery via the LA or with the PA, as measured with the Trendelenburg test [6].

Distinct muscles are disrupted with the LA (the gluteus medius and minimus) and the PA (the gluteus maximus, short lateral rotators, and piriformis), potentially leading to different patterns of muscle weakness [42]. In the current study, surgical trauma was quantified by measuring CpK levels, and the PA group reported lower serum levels after surgery than the LA group. Muscle injury is a result of multifactorial synergy; however, it is generally accepted that surgical dissection of the hip abductors in the PA results in the least severe muscle injury [43].

We next reported that, except for a lower LLD rate for the LA group, other intra- and postoperative complications, including dislocation and reoperation, were comparable between the two approaches. Notably, the majority of patients in both groups (71 and 76 % in the LA and PA groups, respectively) reported leg lengthening; however, the postoperative difference in length was less than the clinically significant value [29], and several studies have shown that up to 10 mm of LLD is well tolerated [44] without impaired function [45]. In our sample, only one dislocation occurred in the LA group. Ji et al. [36] compared the dislocation rate of THA in a prospective randomized trial: 3 dislocations occurred in 97 hips that were subjected to a modified LA, whereas no dislocation occurred in 99 hips subjected to the PA. A systematic review on complications following THA [7] reported that the PA was not associated with an increased dislocation rate compared to that associated with the LA. Skoogh et al. [46] conducted an observational study of the Swedish Hip Arthroplasty Register including 156,979 hips, investigating how the relationship between surgical approach and risk of reoperation has evolved over time and found no significant difference in the risk of reoperation due to dislocation. The authors suggested that increased awareness of the historically higher dislocation risk of the PA, enhanced soft tissue repair and improved surgical techniques for the PA may explain these findings.

Given the retrospective nature of the design of this study, potential bias cannot be excluded. The relatively small sample size for the subgroup analysis may explain our inability to identify significant differences in intra- or postoperative complications between the two groups. The lack of a radiological evaluation and angle calculation of the implant’s position represents a further limitation of the study considering that the PA has been related to a more challenging positioning of the components in comparison to the LA [6]. The prospective nature of the data collection methods, the use of a validated and standardized HRQoL and functional assessment, the statistical reliability, and both the sample size and follow-up, comparable with the largest and longest comparative series available[11, 36, 38, 47], represent considerable strengths of the present study.

## Conclusions

Patients who underwent THA performed with the PA reported greater improvement in HRQoL with lower residual pain, postoperative muscle damage and Trendelenburg signs than those who underwent the LA. No clinically significant differences in functionality and complications were found between the two surgical approaches. Future randomized controlled trials with larger sample sizes may help to confirm the effects of the surgical approaches on HRQoL, functionality and complications following THA.

## Data Availability

The datasets used and/or analyzed during the current study are available from the corresponding author on reasonable request.

## Abbreviations

THA: Means total hip arthroplasty
OA: Osteoarthritis
PA: Posterior approach
LA: Lateral approach
HO: Heterotopic ossification
HRQoL: Health-related quality-of-life
SF-36: Short Form-36 Health Survey
BMI: Body mass index
ASA: American Society of Anesthesiologists
EBL: Estimated intraoperative blood loss
Hb: Hemoglobin
CpK: Creatine phosphokinase
DVT: Deep vein thrombosis
ADL: Activities of daily living
IADL: Instrumental activities of daily living
WOMAC: Western Ontario and Mac Master University
PCS: Physical component summary
MCS: Mental component summary
HHS: Harris Hip score
VAS: Visual Analogue scale
RR: Recovery rate
LLD: Leg length discrepancy
